# Reassessing Rujm el-Hiri: Aerial imagery and stone circles in the proto-historic Southern Levant

**DOI:** 10.1371/journal.pone.0339952

**Published:** 2026-03-18

**Authors:** Michal Birkenfeld, Olga Khabarova, Lev V. Eppelbaum, Uri Berger

**Affiliations:** 1 Department of Archaeology, Ben-Gurion University of the Negev, Be’er-Sheva, Israel; 2 Interdisciplinary Centre for Security, Reliability and Trust (SnT), University of Luxembourg, Luxembourg; 3 Department of Geophysics, Tel Aviv University, Tel Aviv, Israel; 4 Azerbaijan State Oil and Industry University, Baku, Azerbaijan,; 5 Israel Antiquities Authority, Israel; University of South Florida, ITALY

## Abstract

Rujm el-Hiri has long been considered one of the most enigmatic archaeological monuments in the Southern Levant. Variously interpreted as a funerary, ceremonial, or astronomical locale, it has been the centre of multiple studies spanning over more than 50 years. While traditionally viewed as an isolated protohistoric monument, our study reveals it as the most elaborate example of a widespread regional tradition of large, circular basalt stone structures. This study presents a comprehensive regional reassessment of these large circular stone structures in the basalt highlands surrounding Rujm el-Hiri, revealing over 30 previously undocumented examples within a 25 km radius. Utilizing high-resolution satellite imagery, geophysical modelling, and spatial analysis, we document a consistent architectural tradition characterized by concentric and radial basalt walls, often associated with dolmens, tumuli, and field systems. These structures exhibit similarities in design and landscape placement, frequently located near seasonal water sources and integrated within broader agro-pastoral land-use networks. Our findings challenge the view of Rujm el-Hiri as an isolated monument, instead situating it within a wider phenomenon of protohistoric monumental architecture in this region. This expanded dataset provides new perspectives on landscape organization and monumentality in the protohistoric southern Levant. The application of remote sensing techniques proves crucial in overcoming previous survey limitations, revealing a complex and interconnected archaeological landscape hitherto underappreciated.

## Introduction

Monumental architecture has long played a central role in archaeological interpretations of pre- and proto-historic societies, often regarded as a material expression of social complexity, ritual life, and the manipulation of landscape. In the Levant, such expressions are typically associated with the emergence of village societies during the Neolithic [1–5]or with proto-urban developments of the Early Bronze Age (e.g., the monumental Megiddo temple; [6]). Other notable examples also occur, such as the extensive dolmens and dolmen fields of Transjordan, Syria, and northern Israel [7–11].

Located on a basalt plateau in the central Golan Heights, Rujm el-Hiri is one of the most enigmatic archaeological monuments in the Southern Levant. The site, discovered in 1968 by Yizhaki Gal while examining military aerial photographs [12], consists of a central tumulus surrounded by multiple concentric rings of basalt stones. This large structure was labelled the “Israeli Stonehenge”, drawing on the biblical folklore of ancient giants – the ‘Rephaim’ [13]. Despite being the centre of multiple studies spanning over more than 50 years, its date and purpose remain elusive. Over the years, various suggestions have been made regarding its chronology and time of construction, ranging from the Chalcolithic period [14] through the Early Bronze Age [15] to the Late Bronze Age [16], each suggestion referring to different finds recovered at the site. Scholars further debated as to its function, with suggestions ranging from a burial monument, a ceremonial gathering locale, an astronomical observatory, or a complex symbolic marker embedded in a broader cultural landscape ([17–23]; and see discussion below). Still, as no similar structures were identified, the monument’s perceived uniqueness has stood at the base of all, if not most, interpretive attempts. It has long stood in relative isolation, exceptional in its design and scale and lacking clear parallels within its immediate or even wider surroundings. Moreover, the lack of indicative, stratified finds, the absence of clearly associated settlements, and the monument’s formal complexity, as reflected from the archaeological records ([15–17,22–24]; and see below), have limited our ability to place it securely within its regional framework. Although over the years the site and its surroundings were surveyed, excavated, debated, and explored [14,15,20,25], some critical questions regarding the monument’s relationship to its surroundings and to other potential features in its vicinity still remained.

Recent advances in satellite imaging and remote sensing have enabled high-resolution observations of much wider areas, including those inaccessible on foot, either due to harsh terrains or to local geo-political constraints, among other factors. Through multi-seasonal imagery, variations in surface vegetation and light exposure have revealed various archaeological features — linear walls, stone enclosures, circular arrangements, and stone heaps — many of which had not previously been recorded in traditional surveys [26]. In some cases, these features exhibit formal similarities to Rujm el-Hiri, particularly in their circular layout, architectural features, and landscape setting. Their presence raises the possibility that Rujm el-Hiri may not be an isolated construction, but part of a broader architectural pattern embedded within the protohistoric landscape of the region.

If this is the case, the implications are substantial. Such large circular structures may, in fact, be more common than previously believed. This challenges earlier assumptions about the singularity of Rujm el-Hiri and compels us to reconsider our interpretations of it. This article places Rujm el-Hiri not as a stand-alone phenomenon but as a key to understanding a wider tradition of large, circular stone structures in and outside the region. Through a detailed analysis of its landscape context using current remote sensing datasets, we trace repeating spatial patterns that suggest intentionality, planning, and longer life cycles than previously assumed. In doing so, we argue that Rujm el-Hiri should no longer be treated as an archaeological anomaly, but as part of a larger and previously under-recognized phenomenon, one that offers fresh insight into the strategies of protohistoric societies in the region. Rather than an isolated construction, Rujm el-Hiri may have served as one node in a chrono-spatial constellation of related sites, constructed and utilized by agro-pastoral communities navigating the environmental and social spheres.

## Background and previous research

**Fig 1:**
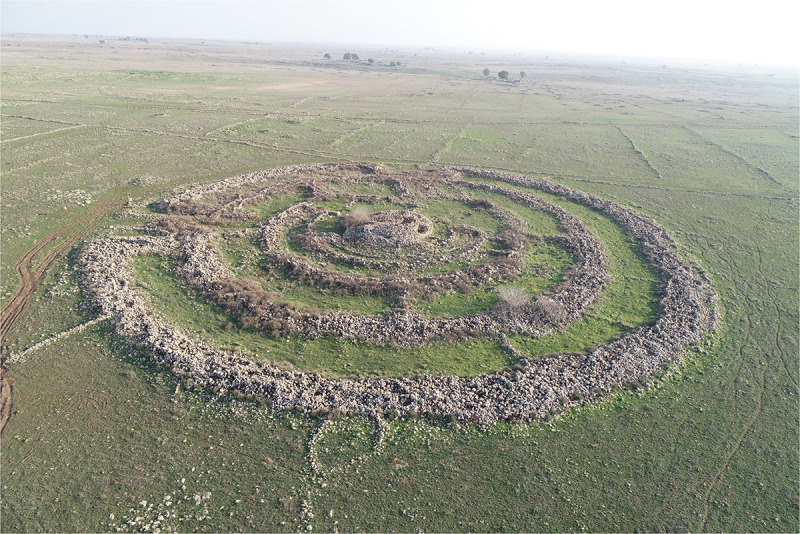
Aerial photo of Rujm el-Hiri, view to east. Photograph by Y. Shmidov and A. Wiegmann.

Rujm el-Hiri has been the focus of intermittent archaeological attention, including excavation, survey, and speculative interpretation [14–17: site 115,^19^,^21^,^22^,^23^,^27^]. The site consists of a central cairn, approximately 5 meters in height and approximately 20 meters in diameter, surrounded by four concentric ring walls, made of basalt stone, some standing over 2.5 meters high and 3.5 meters wide. Two entrances, one to the northeast and one to the southeast, break the continuity of the rings, and radial walls connect the circles at irregular intervals. The total diameter of the structure exceeds 150 meters, making it one of the largest protohistoric stone circles in the region (Fig 1).

Interpretations of Rujm el-Hiri’s function have varied widely. Some scholars have emphasized its funerary aspects, pointing to the central tumulus as a possible extravagant burial of a leader/important persona [18], or suggested that the Rujm was a regional gathering locale, with ritual or ceremonial functions, for otherwise scattered local tribes [19,20]. The Rujm’s defensive properties have also been debated, due to its substantial construction [27]. A particularly influential line of interpretation has emphasized its astronomical alignments, with the northeast entrance in particular thought to correspond to the summer solstice sunrise [21]. Excavations have yielded few datable finds or definitive evidence of funerary use [22,23,28].

More recently, it was shown that correlations between the site’s architecture and ancient celestial events must be reconsidered in light of the site’s own reorientation, not just the evolving sky [26]. Paleomagnetic and tectonic data have indicated that the entire region, along with the archaeological site embedded in it, has undergone gradual but measurable rotational movement over the past four millennia. This means that the site did not remain fixed in orientation relative to the geographic or celestial coordinate systems but rotated together with the surrounding bedrock as part of a broader tectonic process. As a result, the current orientation of features such as radial walls and entrance axes no longer reflects the directions in which they were originally constructed [26]. The displacement, amounting to several meters, is significant enough to invalidate earlier interpretations that proposed intentional astronomical alignments. Even reconstructions that attempt to account for changes in the sky due to precession or other celestial dynamics fall short if they assume the site itself remained geostationary. Since both the sky and the ground have shifted relative to each other, any attempt to project modern measurements backward in time without correcting for tectonic rotation introduces a certain bias.

**Fig 2:**
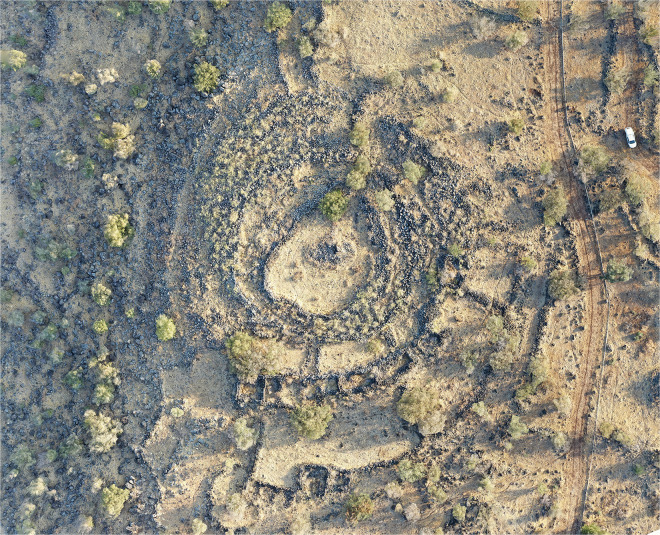
Orthophoto of Khirbet Bteha. Created by A. Kleiner.

**Fig 3:**
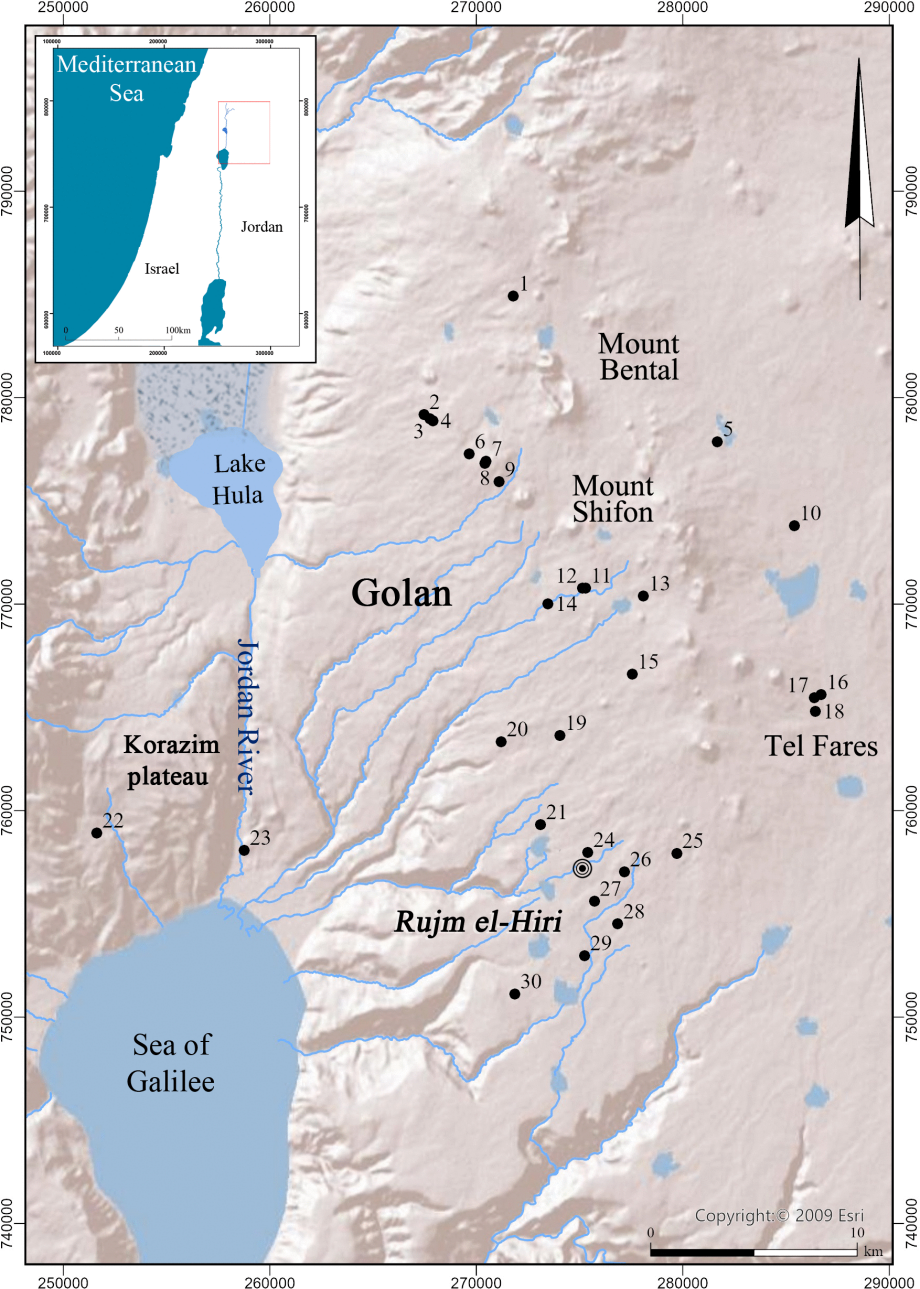
Map showing location of identified circle sites, as well as Rujm el-Hiri and other locales mentioned in the text. Created by U. Berger and A. Shapiro. Base map image is the intellectual property of Esri and is used herein under license. Copyright 2025 Esri and its licensors. All rights reserved.

A longstanding challenge in interpreting Rujm el-Hiri has been the near lack of clearly associated sites of similar design or scale. Traditional field surveys [16–18,25,29,30] failed to identify close formal analogues, reinforcing the notion that the monument was unique. One site that has been mentioned in the literature as bearing similarities to Rujm el-Hiri is the site of Khirbet Bteha, located north of the Sea of Galilee, ca. 16 km west of the Rujm (Fig 2) [14,31]. Situated on the eastern bank of the Jordan River, the structure at Khirbet Betha is similar to the Rujm in shape, comprising at least three large concentric walls, approximately 70 m in diameter. Yet, this intriguing site has never been systematically studied and is hardly mentioned in the literature. This apparent absence of formal analogues seems to reflect methodological limitations rather than an historical or archaeological reality. The emergence of remote sensing techniques, particularly high-resolution satellite imagery and AI-assisted pattern recognition, is beginning to change this picture. Recent studies have identified several large, circular stone features within a 25 km radius of Rujm el-Hiri (Fig 3). Some of these structures exhibit recurring formal elements, such as concentric rings or thick circular walls, among other components. This growing body of data invites a re-evaluation of Rujm el-Hiri, which, instead of standing alone, may represent the best-preserved example of a broader architectural phenomenon. This shift in perspective raises new questions and offers a broader framework for understanding Rujm el-Hiri, potentially shedding light on the larger issue of monumentality in the proto-historic Levant.

## Materials and methods

This study is part of a larger project, focused on the application of Remote Sensing methods in archaeological surveys. It employs an integrated methodological approach that combines high-resolution remote sensing, geophysical modelling, and comparative spatial analysis [26,32]. A part of this project focuses on the archaeological landscape of the Golan Heights in general, and that of Rujm el-Hiri in particular, identifying a dense but fragmented distribution of archaeological remains, including dolmens, tumuli, and other stone heaps, as well as field walls and enclosures [26]. Among these, twenty-eight examples of large circular stone structures stood out. The primary goal of the specific endeavour presented here was to identify, classify, and contextualize these surprising structures within the landscape surrounding Rujm el-Hiri and to evaluate their spatial, morphological, and environmental patterns. The names of the sites appearing in this article are according to the Survey of Israel [32] and their appearances in the maps of Syria and Lebanon, drawn by the Service Géographique des F.F.L [33,34].

### Remote sensing data sources

High-resolution satellite imagery acquired over a two-decade span (2004–2024) formed the core of the remote sensing dataset. The dataset incorporated images from multiple high-resolution platforms (Google Earth Pro, CNES/Airbus, and the Pleiades satellite constellation). Resolutions varied, ranging from 2 m in multispectral bands to 0.5 m in the panchromatic band. Initial visual analysis of the study area was conducted using Google Earth Pro, which provided a contextual overview with imagery primarily sourced from Maxar Technologies and CNES/Airbus. Following this preliminary examination, all subsequent spatial processing, image restoration (where necessary), and quantitative analysis were performed using ESRI’s ArcGIS Pro 3.4.3 (https://www.esri.com/en-us/arcgis/products/arcgis-pro/overview). Within ArcGIS Pro, raster processing and multi-temporal image overlay were executed using the ArcPy Python package, which provides full access to the Spatial Analyst and Image Analyst toolsets. ArcPy enabled the combination and overlay of multiple multi-band (wave-band) satellite images through tools such as Composite Bands, Raster Calculator, and other local raster operations essential for handling temporal stacks of imagery. The ability of the ArcGIS software supported by ArcPy’s precise geoprocessing functions to manage spatial data was important for the temporal layering and overlay of images captured during different seasons and years. This multi-temporal approach was used for enhancing the visibility of subtle landscape features and faint archaeological traces that were often obscured by shadow, seasonal vegetation, or dryness [35,36]. Through overlapping layers, pixel-based raster operations, and adjusting transparency, poorly visible parts of objects across the landscape were effectively revealed and allowed an identification of the objects described below.

### Feature classification

Initially, identified archaeological features were manually classified into typological categories based on morphology: (1) large stone circles of over 50 m, (2) radial enclosures, (3) linear field walls, (4) “flower-like” enclosures, and (5) tumuli or stone-heaps. Among these, large stone circles with diameters ranging from approximately 50–250 m and exhibiting concentric or radial planning were selected for closer analysis. Coordinates and measurements were extracted from the highest-resolution imagery available.

### Landscape context and environmental reconstruction

The region occupies a volcanic plateau that rises markedly above the adjacent lowlands, with an average elevation of roughly 1000 m asl. Distinctive basaltic formations, products of ancient volcanic activity, have shaped both the landscape and the local soil composition. The terrain comprises a series of volcanic hills interspersed with valleys, where erosional and sedimentary processes have created fertile plains rich in minerals, supporting various vegetation (see below) as well as agricultural activities [37,38]. Numerous springs and streams sourced from plateau aquifers sustain both ecosystems and human communities, while drainage channels route much of this water towards the Jordan River and the Sea of Galilee [39]. The region features a diversity of vegetation types, from mixed oak and pine forests to expansive grasslands, fostering rich biodiversity adapted to these varied environments [40]. The area’s proximity to the Dead Sea Transform fault system renders it seismically active, a condition further intensified by the influence of a major deep geodynamic structure [41, 42].

Topographic data and elevation models were used to map hydrological features, particularly seasonal stream channels and paleo-depressions likely to have held water in the early to mid-Holocene. Spatial analysis included proximity to water sources, elevation profiles, and slope. The goal was to determine whether the placement of circular structures was random or clustered near certain environmental thresholds. All spatial analysis was conducted using ESRI’s Arc GIS Pro 3.4.3. A 10 m cell-size DEM was used as the basis for analysis. Two independent tools were used to reconstruct seasonal streams, both available via the Spatial Analyst toolset. These include the Derive Stream as Raster tool as well as the Flow Accumulation tool. The distance between sites and the identified streams as well as additional proximity information was then calculated using the Near tool.

## Results

Twenty-eight large circular structures were identified in the current analysis, all within a 25 km radius from Rujm el-Hiri (Table 1; Fig 3). While only two have been recorded in the past (i.e., Circle #11, Wadi es-Sqat,and #23, Khirbet Bteha; [14,31,43] and see below), the vast majority represent new additions to the region’s archaeological record. These sites all follow a similar design, i.e., large circular structures, usually more than 50 m in diameter, constructed of local basalt fieldstones. While few are simple in their plan, consisting of a single round wall, most show a more intricate design, incorporating concentric circles, connecting walls, and/or other features.

**Table 1:** All circle sites identified, their location, and characteristics.

#	Latitude	Longitude	Diameter	Distance to nearest stream	Concentric walls	Perpendicular/ connecting walls	Cluster
1	33° 9’30.42“N	35°45’55.34“E	~ 55m	~ 130m	2	–	+
2	33° 6’24.86“N	35°43’7.35“E	~ 65m	~ 350m	2	–	+
3	33° 6’18.15“N	35°43’18.00“E	~ 65m	>500m	2?	?	+
4	33° 6’14.69“N	35°43’23.88“E	~ 60m	>500m	3?	–	+
5	33° 5’39.25“N	35°52’15.17“E	~ 75m	No Data	2	–	–
6	33° 5’22.35“N	35°44’31.32“E	~ 98m	~ 350m	2?	–	–
7	33° 5’10.66“N	35°45’3.13“E	~ 92m	>1000m	–	+	+
8	33° 5’7.64“N	35°45’0.60“E	~ 72m	>1000m	–	+	+
9	33° 4’38.22“N	35°45’27.36“E	~ 250m	~250m	2?	+	–
10	33° 3’26.79“N	35°54’37.51“E	~ 72m	~85m	?	–	–
11	33° 1’50.68“N	35°48’7.14“E	~ 73 m	~50m	2	–	+
12	33° 1’50.32“N	35°48’2.00“E	~ 95 m	~35m	2	–	+
13	33° 1’37.67“N	35°49’55.54“E	~ 92 m	~40m	2		
14	33° 1’26.29“N	35°46’56.95“E	~ 95 m	~35m	2/3?	+	–
15	32°59’34.69“N	35°49’33.33“E	~ 95 m	~45m	–	–	–
16	32°59’0.58“N	35°55’25.63“E	~ 55 m	~40m	2+	–	–
17	32°58’56.13“N	35°55’12.63“E	~ 80 m	~180m	2	+	–
18	32°58’34.47“N	35°55’14.52“E	~ 75 m	~350m	2	?	–
19	32°57’59.08“N	35°47’17.66“E	~ 65 m	~100m	–	–	–
20	32°57’49.77“N	35°45’28.28“E	~ 90	~200m	2	+	?
21	32°55’38.99“N	35°46’41.36“E	~ 80 m	~65m	2	–	–
22	32°55’28.95“N	35°32’54.18“E		NoData			–
23	32°55’0.84“N	35°37’28.85“E	~ 105 m	~100m	4?	+	–
24	32°54’55.03“N	35°48’8.97“E	~ 62m	~200m	2	–	–
25	32°54’52.34“N	35°50’54.40“E	~ 80	~40m	2?	+?	–
26	32°54’24.20“N	35°49’16.64“E	~ 52 m	~100	–	?	–
27	32°53’37.93“N	35°48’20.45“E	~ 90 m	~250m	?	?	–
28	32°53’2.12“N	35°49’3.82“E	~ 40 m	~60m	–	–	–
29	32°52’12.64“N	35°48’2.14“E	~ 53 m	~200m	–	–	–
30	32°51’13.02“N	35°45’51.16“E	~ 100 m	~300m	2	?	–

**Fig 4:**
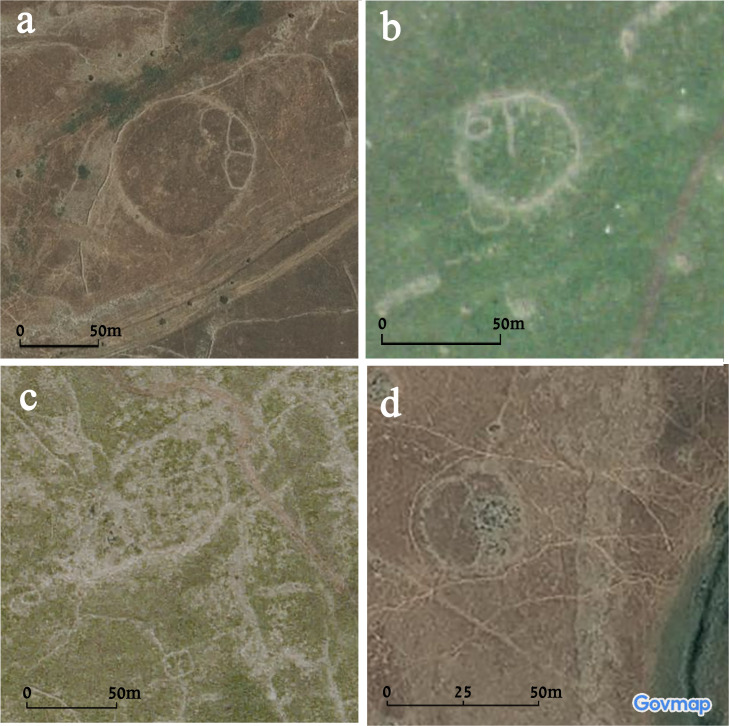
Aerial views of the four ‘simple’ circles: a) Circle 15, b) Circle 29, c) Circle 19, d) Circle 28. Aerial imagery provided by the survey of Israel (MAPI)- used with permission. *Created by M. Birkenfeld and U. Berger*.

Simple, single-wall sites include four examples (Circles 15, 19, 28, and 29; Fig 4). These include some of the smaller examples, with a diameter of approximately 50 m (Circles 28 and 29), as well as larger ones (e.g., Circle 15, with a diameter of approximately 95 m). These circles are composed of a single circular stone-built wall, approximately 2–3 m wide. Later-period installations and walls can also be identified, at times abutting the circular wall or cutting it, postdating the circles. These include small enclosures, field walls, as well as tumuli (e.g., Circle 28; Fig 4: 4D).

**Fig 5:**
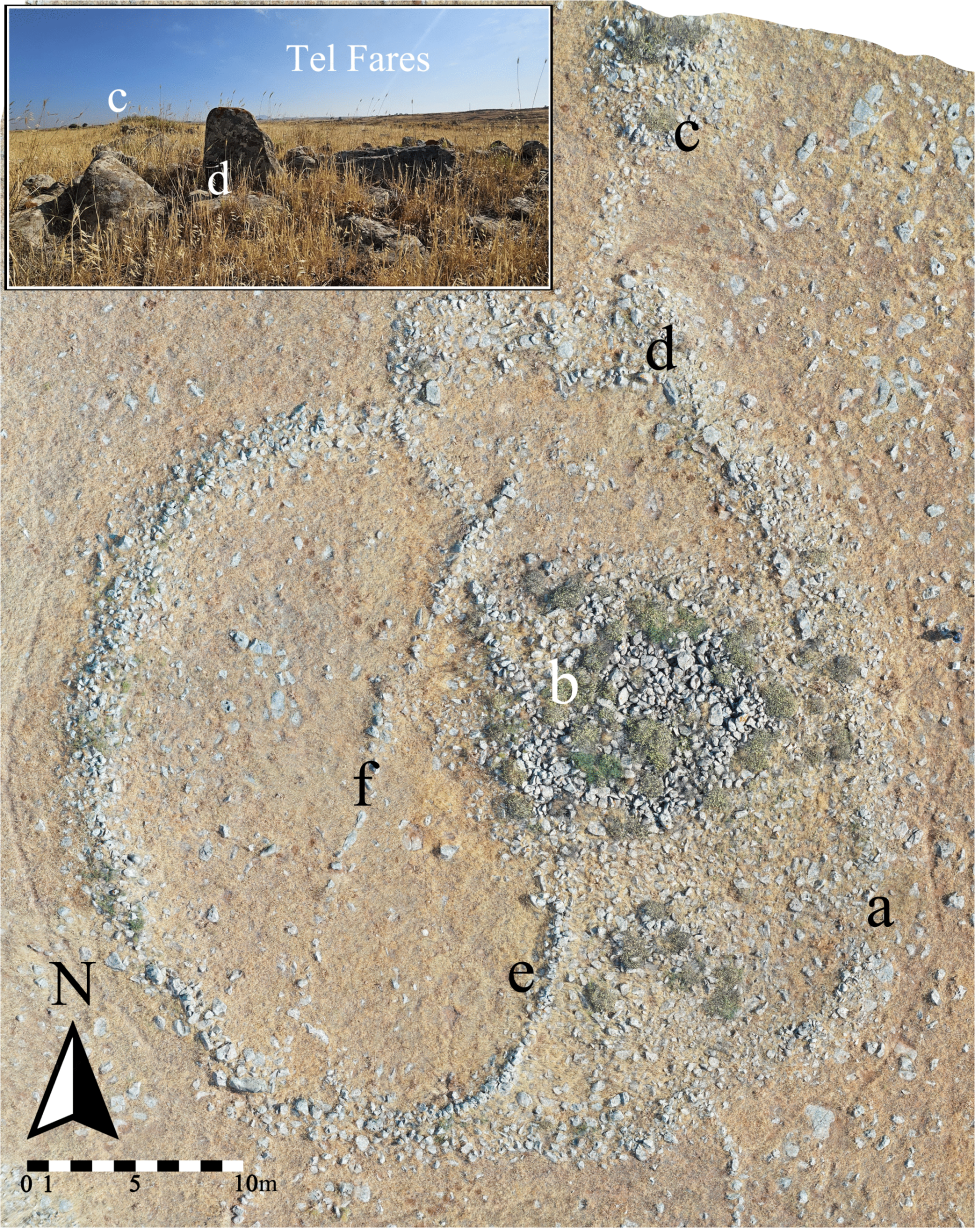
Aerial view of Circle 28 (a), showing location of Tel Fares, as well as the two dolmens (b & c), standing stone (d), and two post-dating walls (e & f). *Photography by A. Kleiner. Graphics by U. Berger*.

Circle 28 is an example of such a simple circle; it consists of a one-meter-wide bifacial stone-built wall, approximately 40 m in diameter (Fig 5: 5a). A single dolmen, in poor preservation, is situated inside the circle (Fig 5: 5b), and another is located to its north (Fig 5: 5c). A standing stone located in its northeastern part (Fig 5: 5d) faces Tel Fares (Fig 5), one of the largest volcanic mounds in the region and one of the ‘physical elements’ associated with Rujm el-Hiri (sensu [21: 490]). Later constructions include two straight field walls (Fig 5: 5e-5f) built within the circle. One of the field walls (e) cuts the circle into two uneven enclosures and builds upon the ruins of the inner dolmen, hence later to it. The other field wall (f) crosses the circle from south to north, ‘cancelling’ the former field wall (e).

**Fig 6:**
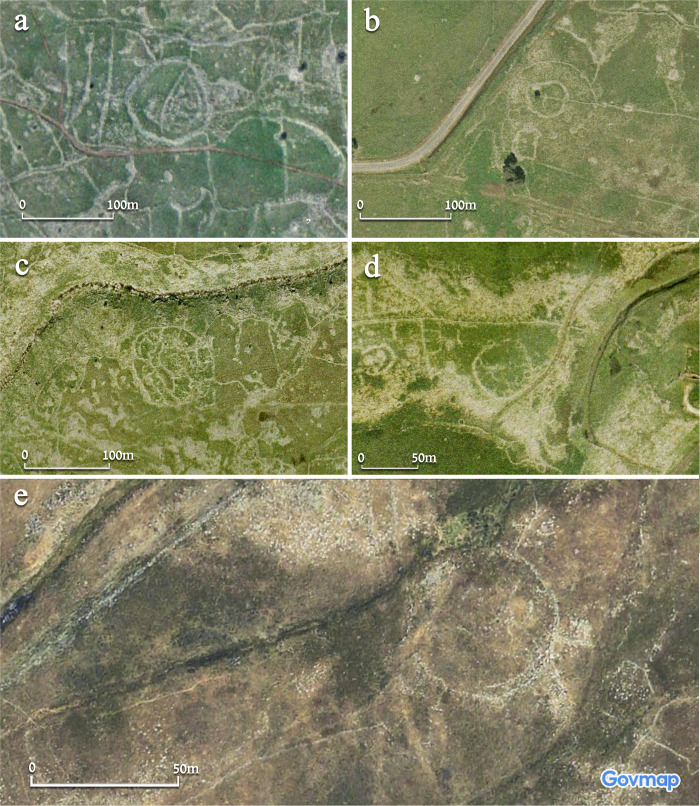
Aerial views of complex circles: a) Circle 6, b) Circle 30, c) Circle 14, d) Circle 24, e) Circle 26. Aerial imagery provided by the survey of Israel (MAPI)- used with permission. *Created by M. Birkenfeld and U. Berger*.

Most circles (n = 20) are comprised of two (and at times three) concentric walls (Circles 1, 2, 3, 4, 5, 6, 11, 12, 13, 14, 16, 17, 18, 20, 21, 23, 24, 25, 26, and 30; Fig 6: 6a-6b). Their diameter ranges between 52 and 105 m (with an average of 82 m and a median of 70 m), but they all follow a similar plan. A good example is Circle 30. This circle is comprised of two concentric walls: The outer wall, approximately 100 m in diameter, and the inner wall, approximately 40 m in diameter. Both walls are made of basalt fieldstones and are ca. 2 m wide. At least two postdating walls seem to cut Circle 30, connecting it to the broader landscape of field walls and agricultural plots. A semi-circular enclosure sits on top and postdates the circle on its southern side (Fig 6: 6b). Another example is Circle 6. Here, as well, aerial photographs have indicated the existence of two circles: The outer, approximately 100m in diameter, is well-preserved and quite wide (approximately 4–5 m). The inner wall is thinner, approximately 2 m in width, but its preservation is lesser (Fig 6: 6a). It can be easily delineated from the east, south, and southwest, but its northwestern part is missing. It is quite large as well, with a reconstructed diameter of approximately 70 m. A long wall transects the circle from north to south, clearly postdating it. From the centre of this circle, there are clear views of Mount Hermon to the north (the highest landmark in the region), and Mount Bental/Tell el Arâm to the to the East. A large dolmen field was recorded on a basaltic terrace immediately west of the circle.

Another large group of circles exhibits not only concentric walls, but also shorter linear walls, perpendicular to the outer circular wall, and at times connecting it to an inner wall and/or other features situated in the centre of the structure (n = 9, Circles 7, 8, 9, 14, 20, 23, 24, 25, and 26; Fig 6: 6c-6e).

Circle 14, located ca. 40 m south of a small perennial stream leading to the Zavitan/ Zouaïtinaïne river, is comprised of two concentric walls, the largest of which ranges between 93 and 105 m (in its east-west and northwest-southeast axes, respectively). A third radial element, clearly seen in the image, could represent a third, wider concentric wall in poor preservation. the two clearly recognizable circular walls, approximately 2 m in width, are connected by short, linear walls that transect the circular rings into sections, in a similar manner to those of Rujm el-Hiri, although much less regular or symmetrical. Several field walls adjoin the structure from outside, connecting it to a line of large enclosures, some abutting the structure itself though most probably postdating it (Fig 6: 6c).

Other examples, smaller in scale but similar in form also exist. Circle 24 (Fig 6: 6d) consists of a wide stone-built wall, ca. 2–3 m in width, and ca. 62 m in diameter. It shows evidence of a thinner internal concentric wall, ca. 1–15 m in width, connected to the outer wall by at least two linear walls. At least three smaller chambers can also be identified, abutting the inner face of the outer wall to the north-east, east, and west. These small chambers, ca. 5–8 m in diameter, most probably represent a later addition to the structure.

An interesting variation can be seen in Circle 26, which, like Circle 14 and 24, comprises a large, round stone-built wall (ca. 1 m in width and ca. 52 m in diameter) and several shorter perpendicular walls connecting the outer wall to a round feature or circular cell, situated within it, slightly off-centre (Fig 6: 6e). Other chambers can also be identified, abutting the outer wall, both from within and outside the structure, possibly representing later additions. A road, running from southwest to northeast, cuts and postdates the structure.

**Fig 7:**
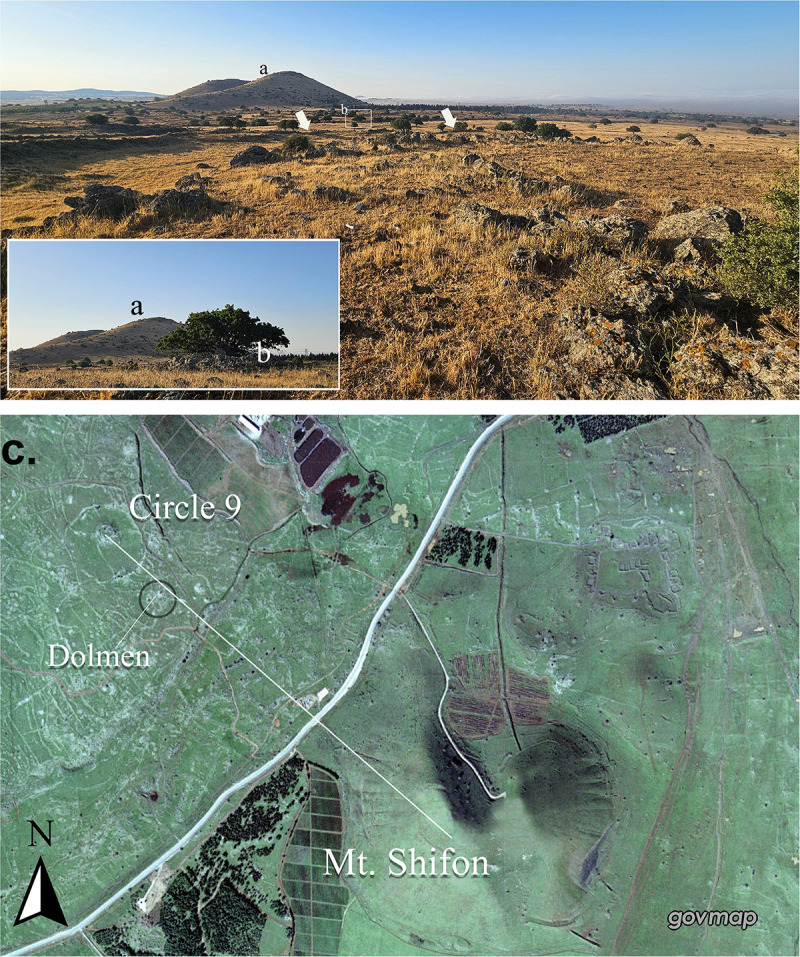
Ground and aerial views of Circle 9, showing Mt. Shifon/Tell Abou Hanzîr (a) and the dolmen located outside the circle (b). Aerial imagery provided by the survey of Israel (MAPI)- used with permission. *Created by U. Berger*.

This variation is echoed by the largest structure identified to date, Circle 9. This structure, ca. 250 m in diameter, is located ca. 300 m from a small tributary of the Gilbon/Jalabina stream. Similarly to Circle 26, it exhibits a large, round stone-built wall, ca. 5 m in width, that was constructed around a round feature, this time a small volcanic cone. Four thinner linear walls, approximately 1 m in width, connect the outer circle to the cone at its centre. It is interesting to note that from the circle there’s a clear view of Mount Shifon/Tell Abou Hanzîr, a prominent marker in the landscape, standing tall at 978 meters above sea level (Fig 7: 7a). A single dolmen (Fig 7: 7b), with a burial chamber, was constructed along the southeast axis between the peaks of Shifon/Tell Abou Hanzîr and the cone in the centre of Circle 26. The dolmen is also associated with a tumulus and a ring-shaped stone wall (ca. 14 m in diameter).

**Fig 8:**
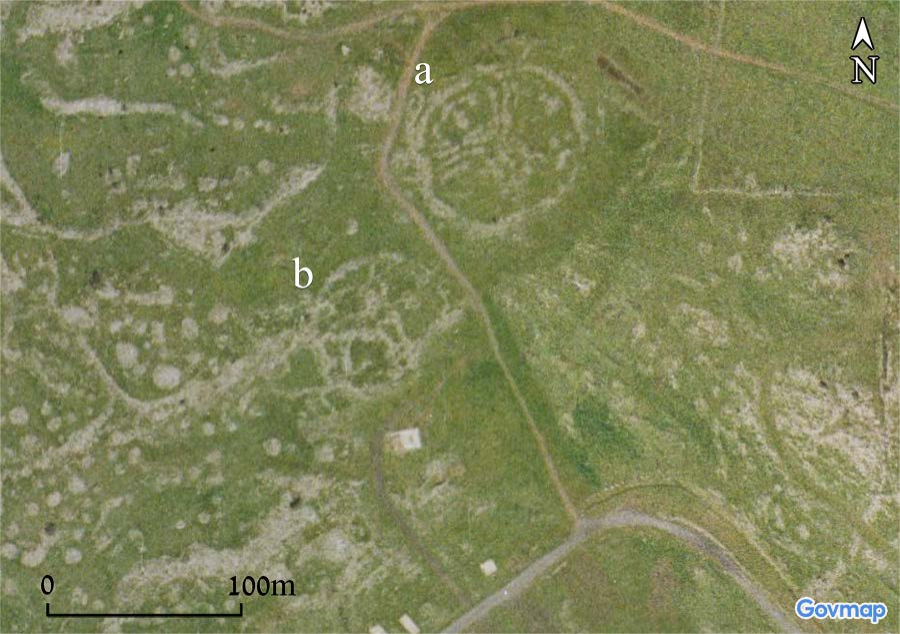
Aerial photo of Circles 7(a) and 8(b). Aerial imagery provided by the survey of Israel (MAPI)- used with permission. *Created by M. Birkenfeld and U. Berger*.

Another unique feature can be found in Circles 7 and 8. These two adjacent structures (and see discussion below) exhibit relatively large outer circles (92 m and 72 m in diameter, respectively), with four double perpendicular linear walls intruding from the outer wall inward. This is clearer in Circle 7, which seems to be better preserved than Circle 8 (Fig 8).

**Fig 9:**
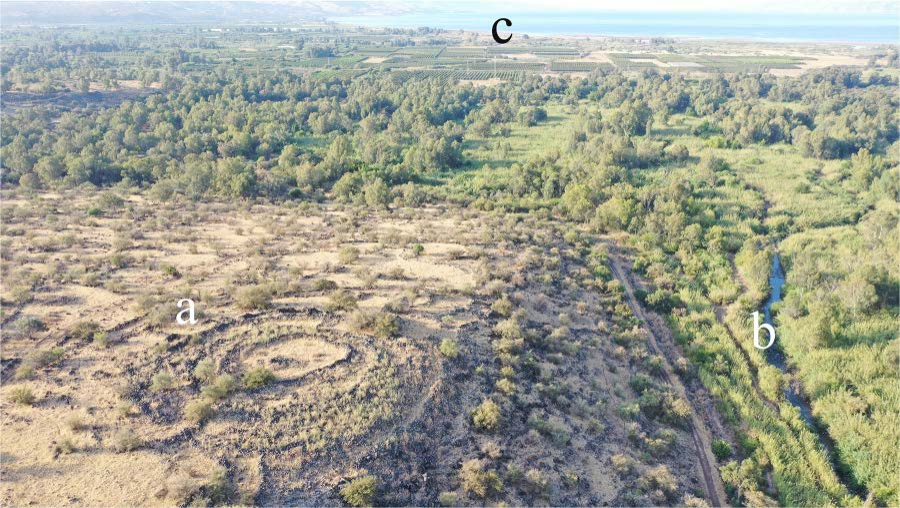
Aerial view of Khirbet Bteha, showing the circle (a), the Jordan River (b), and the Sea of Galilee (c). Created by U. Berger.

One of the most impressive examples within this group is Circle 23, also known as Khirbet Bteha (Figs 2 and 9). The site, located on the eastern bank of the Jordan River, was discovered by Epstein and Gutman during the survey of the Golan in 1967/8 and was partly excavated in 1976 by Epstein [31]. No indicative pottery was found in this excavation, and no specific date was thus suggested for the site. It consists of at least three concentric walls (55, 30, and 11 m in diameter) connected by several short perpendicular walls, creating a line of cells visible mainly in the south/southeast part of the structure. In the centre of the circles, a stone heap containing several large basalt slabs might hint that a dolmen used to stand on site.

As mentioned above, in the context of Circles 7 and 8, some circles appear in tight clusters, in proximity and, at times, in clear association with one another. This is clear in the case of Circles 7 and 8, which are situated approximately 25 m apart from each other. Four other such clusters were identified, including Circles 1, 2, 3, 4, 7, 8, 11, 12, 16, 17, and perhaps also Circle 20.

**Fig 10:**
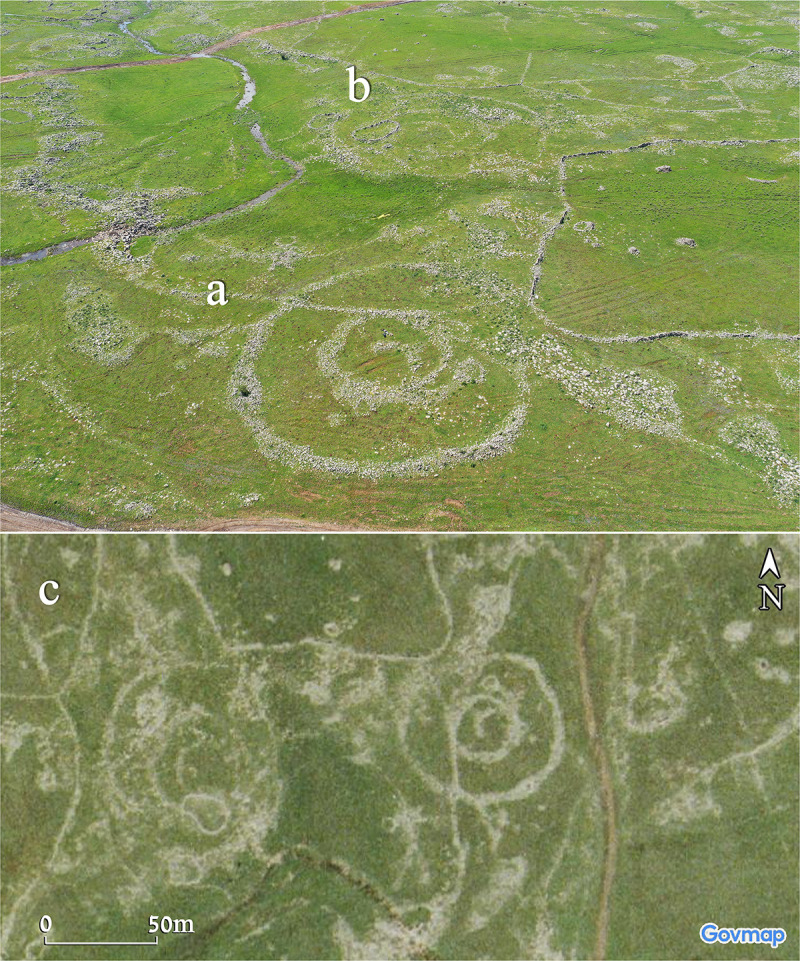
Aerial views of Circles 11(a) and 12 (b). Upper photo by A. Wiegman. Aerial imagery provided by the survey of Israel (MAPI)- used with permission. *Prepared by M. Birkenfeld and U. Berger*.

One of the clearest examples, similar to Circles 7 and 8, are Circles 11 and 12, also known as the site of Wadi es-Sqat. These two adjacent circles, situated on the northern banks of Zavitan/Zouaïtinaïne ravine, have both been recently analysed using a combination of image reconstructions and neural network image recognition [44]. One of these circles (Circle 11) was identified and surveyed in the 1990s ([43]: Site 49). It is comprised of two concentric round walls, the outer one approximately 73 m in diameter, and the inner one approximately 30 m in diameter. Both walls are constructed of medium-sized fieldstones, approximately 3 m wide, and are preserved to a height of approximately 0.5 m (Fig 10). A third, straight wall was also described within the central circle by Hartal, who suggested an Early or Middle Bronze Age date to the site based on surface distributions of lithic artefacts. A later period wall can also be seen in the aerial image, cutting the site from north to south, as well as a small, circular cell or chamber, ca. 8 m in diameter. Circle 12, located ca. 50 m west of Circle 11, shows a remarkable similarity to Circle 11, though it is much less preserved, explaining perhaps the fact that, as high resolution as Hartal’s foot survey was, and although he made use of aerial photography, this site remained unnoticed. It consists of two, perhaps three, concentric walls, with the outer one being approximately 90 m in diameter, and the inner ones 50 and 30 m in diameter (Fig 10). Here, several large stone heaps can be identified constructed on top of and clearly post-dating the original structure. Two large circular walls, most probably representing later-period enclosures, can also be identified in the southern part of the structure. Our previous analysis has highlighted the circular and somewhat spiral nature of both circles, suggesting a chrono-stratigraphic ordering of the various visible features [32, p. 7, Fig 5].

**Fig 11:**
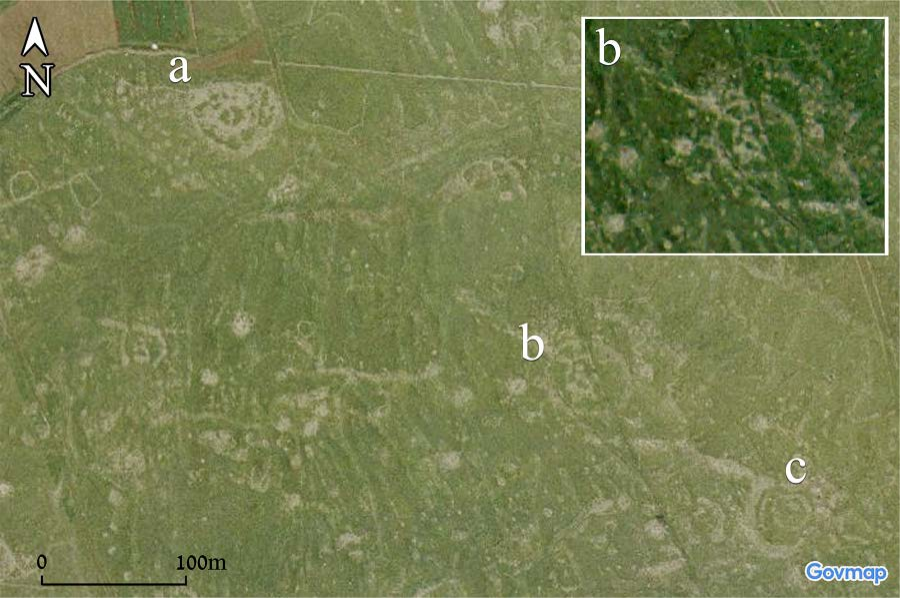
Aerial view of Circles 2 (a), 3 (b), and 4 (c). Aerial imagery provided by the survey of Israel (MAPI)- used with permission. *Created by M. Birkenfeld and U. Berger*.

A third, tripartite cluster is represented by Circles 2, 3, and 4 (Fig 11). These three circles are quite similar to one another, both in form and in scale, each consisting of at least two concentric circles, the outer wall 60–65 m in diameter and the inner wall ca. 30 m in diameter. They appear to be aligned on a northwest-southeast orientation, with Circle 2 located approximately 270 m from Circle 3, which in turn is approximately 120 m from Circle 4. Both Circles 3 and 4 exhibit a long outer wall, protruding from each circle towards the northwest, possibly connecting the circles together.

**Fig 12:**
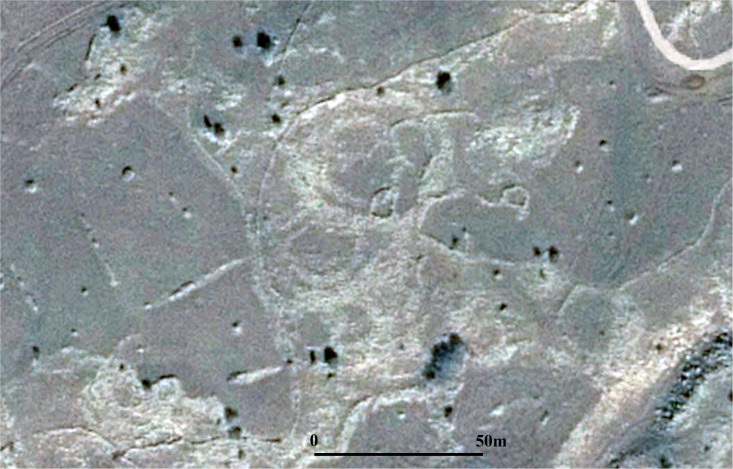
Aerial view of Circle 1. Aerial imagery provided by the survey of Israel (MAPI)- used with permission. *Created by M. Birkenfeld*.

Another possible tripartite cluster is represented by Circle 1, comprised of a series of three superimposed circles (Fig 12); the largest, uppermost circle is ca. 55m in diameter, while the other, supposedly earlier two, are smaller in area (ca. 45m and 30m in diameter), and exhibit lesser levels of preservation. Still, they all seem to share a similar plan, consisting of at least two concentric walls. Lastly, Circles 16 and 17 are located ca. 280 m one from the other. Circle 16 presents an example of a poorly preserved round structure. While only one concentric wall, ca. 55 m in diameter, can be clearly defined, the remains of another, outer wall can be suggested by partial remains to the southeast. A third, round inner wall can also be identified, preserved to a length of ca. 25 m. Circle 17 exhibits two concentric stone-built walls, ca. 2–3 m in width and ca. 80 and 35 m in diameter. At least three thinner perpendicular walls are also visible, connecting the two circles from several directions. A fourth wall appears to be a later addition, lying on top of and cutting into the outer wall to the east. Other features are more complex to decipher due to the poor preservation of the structure and the quality of the available imagery. It is interesting to note that in almost all clusters, one of the circles exhibits much better preservation than the other/s. This is especially true for Circles 1 and 11–12 but is also recognizable in Circles 7–8 and 16–17.

**Fig 13:**
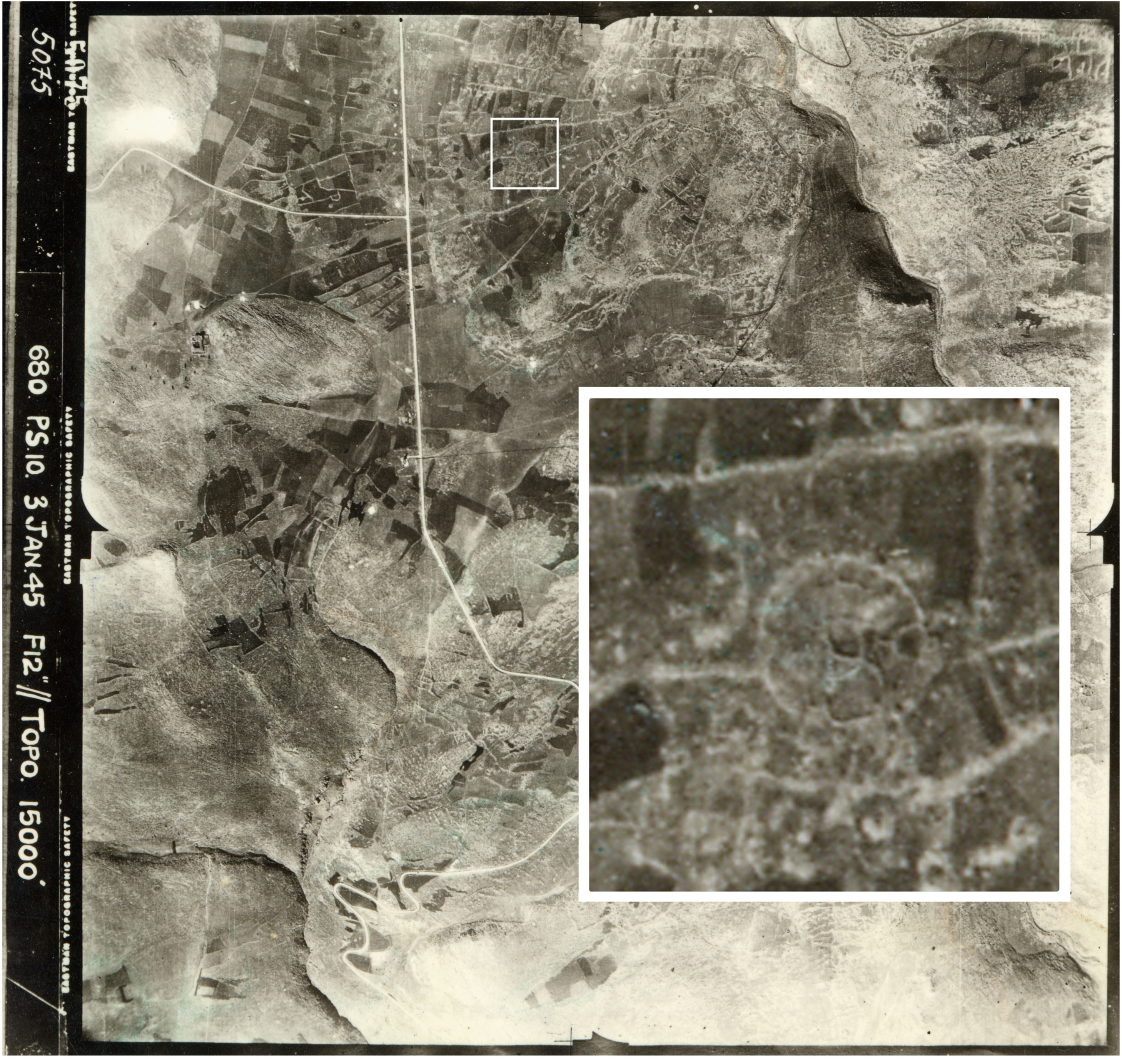
Aerial photo of the Amiad Circle; British Airforce, Aerial imagery provided by the survey of Israel (MAPI)- used with permission. *Created by U. Berger.*

## Discussion

If we summarise thus far, the sites described above vary in size, ranging from approximately 50–250 m in diameter, but seem to share a suite of characteristics, including: a) Circular or near-circular, thick walls, ca. 2–3 m in width, built of medium to large local basalt fieldstones; b) Internal features, including radial or concentric walls, and linear partitions, usually thinner than the outer walls, ca. 1–2 m in width; and c) Their preservation is typically poor, and as seen, for example, in circles 12, 14, 19, 28, or 30, as there are numerous signatures of the secondary use of their material, and of later period/s features abutting the internal and outer walls, at times constructed above them and cutting them. Unlike Rujm el-Hiri, most of these sites have not been excavated or even documented in field surveys. Given their lesser preservation and the fact that much of their construction materials have been used and reused in later periods, limits their visibility significantly. Even from the air, their visibility is usually limited to dry-season satellite passes or low-angle sunlight conditions.

A notable characteristic of these sites is their location within the landscape. Most identified large circle clusters described above occur on the extensive plateau above and east of the large river drainage systems running from the Golan Heights westwards towards the Sea of Galilee, at elevations between 400–600 meters above sea level. The examples from the Korazim Plateau and Khirbet Bteha represent a different geographical setting, but it is very similar in its landscape to that of the other circles. All sites are situated on gentle slopes or small plateaus adjacent to small natural drainages. No circles were found in lowland or heavily terraced areas. Intriguingly, many of the identified sites, just like Rujm el-Hiri itself, are located within a short distance, if not immediately adjacent to a seasonal waterway (Table 1). Lastly, most sites appear to be integrated within larger field systems or wall networks. An interesting association can be discerned between these circular constructions and dolmens. This is especially apparent in Circles 6, 9, and 28, but is evident elsewhere as well. For example, Khirbet Bteha (Circle 23) is located immediately south of the Had Nes and Wadi ed Dikka dolmen fields ([45,46,47: Sites 360, 361, 362]). This echoes the long-debated association between Rujm el-Hiri and the dolmens surrounding it (the el-Arbain dolmen field; [48]). Many have mentioned the spatial correlation between the two phenomena [14,18,24,25]. At this stage of research, it cannot be determined whether this association represents the result of later activity or whether it is part of the original construction of the circles. In any case, they are part of broader, intertwined, land-use practices. This connection to the wider landscape is also evident in the association of some circles with prominent landscape markers, mainly mountain tops, as mentioned above, also apparent at Rujm el-Hiri itself (e.g., [21: 490–492,^24^]).

It should be stressed that the structures described in this paper in no way represent the entirety of the phenomenon. It can be assumed that other stone circles were scattered across the region. Some may have been destroyed over the years due to agricultural or other modern activities. An interesting example of such a structure can be seen in an aerial photograph dated January 1945, approximately 25 km west of Rujm el-Hiri on the Korazim Plateau (Circle 22; Fig 13). Located west of the Jordan River, the Korazim plateau is considered part of the Upper Galilee, but like the Golan Heights, it is a large basaltic plateau. The large round structure depicted in the photo consists of a large concentric wall, and several cells/chambers can be identified with it, similar to the circles described above. Notably, a 70-meter-wide submerged circular structure was discovered in the Sea of Galilee, approximately 500 meters offshore [49].

This newly identified phenomenon resonates similar, perhaps related phenomena elsewhere in the southern Levant, in Syria and Jordan (e.g., Conder’s Circle [50–52]; but also see [53]). Interestingly, some of these examples (like Conder’s circle) have also been associated with local dolmen fields [10:100]. Other examples, in the wider Mediterranean sphere and even beyond, bring to attention the significance of large, round megalithic monuments, in different landscapes. Examples are many, and include the recently discovered Minoan Papoura Hill circular structure unearthed in June 2024 northwest of Kastelli, Crete (Archaeology Wiki June 12^th^, 2024; https://www.archaeology.wiki/blog/2024/06/12/a-unique-find-for-minoan-archaeology/). This structure, approximately 50 m in diameter, consists of eight concentric walls surrounding a central round chamber. Perpendicular walls segment the structure into smaller chambers/rooms, similar in fashion to those of Rujm el-Hiri and Khirbet Bteha. Other examples include a massive, 1-kilometer-wide circular structure near Wadi Tumilat in modern-day Egypt, identified using spaceborne synthetic aperture radar [54]. Notwithstanding the difference in scale, the striking architectural resemblance of this structure to those presented in our study suggests a potentially wider cultural or functional application of such monumental forms across the broader region.

To summarise thus far, the identification of multiple large circular structures, each measuring tens of meters in diameter, in the basalt landscape of the Golan brings attention to a previously unreported pattern of archaeological occurrences in the region. Apparent similarities can be identified in the geometry, design, and measurements of these sites, as well as in their location within the landscape (both the physical landscape and that of past human activity). These characteristics, together with the architectural motifs of several concentric walls, at times bisected or connected by perpendicular linear walls and the association of the circles with field walls, smaller enclosures, dolmens and tumuli, are all mirrored at Rujm el-Hiri. This may suggest a connection between these sites, including Rujm el-Hiri, possibly implying that they might be related and signify a distinct phenomenon.

If so, this clearly has significant implications for understanding Rujm el-Hiri and the broader cultural landscapes within which it existed. Rather than an isolated case, Rujm el-Hiri may be the most elaborate representative of a now-evident architectural tradition. These circles could have served multiple functions: ritual gathering places, territorial markers, or commemorative monuments. They could be related to herd keeping or represent sites of seasonal aggregation. Their construction near water sources and their integration with field systems hint at a meaningful relationship between them and wider landscape use patterns.

In this respect, our analysis may have implications on former interpretations of the function or functions of Rujm el-Hiri. The recent discovery of tectonic displacement [26] has reinforced scepticism regarding fixed astronomical alignments of Rujm el-Hiri. If the Rujm’s uniqueness within the landscape is questioned, the plausibility of the identification of the site as an observatory is further diminished. This does not preclude symbolic or even celestial orientations, but it calls for greater caution in their interpretation.

### Temporality, visibility, and survey bias

As mentioned above, all sites exhibited clear signs of use and reuse, with clear evidence of the recycling of their building materials and their repurposing during later periods. This affects not only their long-term preservation but also drastically influences their on-ground visibility. In fact, several of the newly identified circles were in areas that were surveyed in the past. The case of Circles 11 and 12 makes a strong example: While Circle 11 was identified and recorded in full during Hartal’s survey [43], Circle 12, just 50 m away, was not identified from the ground. This underscores the importance and potential benefits of remote sensing techniques as tools for archaeological survey. In this case, remote sensing techniques not only expand our spatial awareness but also challenge long-standing biases about where monumental architecture “should” be found.

The reuse and overbuilding of circular structures also hold other implications, more related to their socio-cultural significance: The possibility that circles were re-built in adjacence to older circles suggests that these were not one-time constructions, but sites of long-term significance. They may have operated as mnemonic devices or enduring nodes of spatial identity within the shifting social and ecological landscapes. Though the exact timeframe remains unknown, dependant on our ability (or lack of) to securely date these circles, these associations support the interpretation that they existed within a palimpsest landscape, where monuments were reused, modified, and reinterpreted over time.

## Conclusions

By combining satellite imagery and environmental analysis, this study attempts to reposition Rujm el-Hiri: Long viewed as an almost isolated monument, it emerges from this study as the pinnacle, the most elaborate example to date of a coherent and extensive tradition of large stone circles in the southern Levantine basalt highlands. These circles, identified through remote sensing and contextualized through geophysical and environmental data, invite a reinterpretation of significant protohistoric monuments in the region. Rather than focusing solely on site-specific function, we argue for a landscape-based perspective – one that recognizes these structures as integral to wider social and economic systems.

The work presented here is clearly the first step towards such a wider, landscape-based perspective. Further analysis is of course required, clarifying not only the detailed chronologies and life-stories of these structures, but also correlating them with other elements of the human landscape, such as settlement sites and task-specific sites in the region. The correlation between stone circles and the vast tumuli fields should also be further investigated. Only then will we be able to reach a fuller understanding of these monuments of our shared human past.
